# Blood, ink, and flies: A case report on the house fly (*Musca domestica*) feeding on active tattooing wounds

**DOI:** 10.1007/s00436-026-08792-1

**Published:** 2026-10-21

**Authors:** Patrick Klüber, Niklas Hetz

**Affiliations:** Fraunhofer Institute for Molecular Biology and Applied Ecology IME, Ohlebergsweg 12, Giessen, 35392 Germany

**Keywords:** Wound hygiene, Exsudatophagy, Dermal infection risk, Body art, Mechanical vector, Pathogen transmission

## Abstract

Cases of adventitious feeding on humans by non-hematophagous insects hold significant medical relevance, yet their occurrence during active body art procedures remains undocumented. This case report describes a persistent instance of opportunistic exsudatophagy by the house fly, *Musca domestica* Linnaeus, 1758 (Diptera: Muscidae), during a tattooing session on a healthy 32-year-old male client in Germany. Over a four-hour timeframe, four adult house flies entered the ground-floor studio through doors and windows opened for summer cross-ventilation. The insects exhibited highly persistent landing and feeding attempts directly on the freshly wounded tissue of the right lower leg, actively drinking a matrix of blood, plasma, and tattoo ink. Attraction intensified significantly once large-scale shading expanded the exuding epidermal surface area. Effective mechanical repulsion was heavily restricted by the client’s enforced immobility required to ensure precise tattoo application. Following a routine aftercare protocol, the lesion healed completely without secondary bacterial infection. This report highlights a neglected hygienic hazard where the saprophagous and coprophilic ecology of synanthropic filth flies poses a mechanical vector risk for dermal pathogen inoculation, emphasizing the necessity of simple vector control strategies in tattooing facilities.

## Introduction

Tattoos are becoming increasingly popular as a highly prevalent form of decorative body art. By injecting pigment granules and additive formulations into the dermis, permanent skin pigmentation is achieved. Mechanistically, this process represents a controlled, large-scale breach of the skin barrier by specialized needles. This structural trauma triggers a natural local inflammatory response and, consequently, results in the continuous release of wound exudates such as blood, interstitial fluid, and plasma. Therefore, strict aseptic hygiene protocols are necessary to minimize the risk of bacterial, viral, fungal, and parasitic infections within fresh tattoo wounds (Bălăceanu-Gurău et al. 2024).

However, environmental sources of contamination extending beyond those controlled by standard institutional hygiene measures – such as skin disinfection, single-use sterile equipment, sterile ink, and standard post-procedural aftercare (Dieckmann et al. 2016) – have received significantly less scholarly attention to date. One potential source of such external contamination is direct contact with synanthropic insects. Several insect species – spanning diverse non-hematophagous taxa that opportunistically feed on host substrates like sweat (sudophagy, e.g., *Cethosia cyane* Drury, 1773 (Lepidoptera: Nymphalidae), tears (lachryphagy, e.g., *Lisotrigona cacciae* Nurse, 1907 (Hymenoptera: Apidae), or wound fluids (*Hypochrosis pyrrhophaeata* Walker, 1863 (Lepidoptera: Geometridae) – have been reported to target living humans, with a representative selection of these documented interactions compiled by Klüber and Hetz (2026). This adventitious behavioral pattern is generally termed exsudatophagy (Klüber and Hetz 2026). The epidemiological relevance of these accidental host-vector interactions depends heavily on the specific ecology and feeding habits of the individual insect species (Belluco et al. 2023).

In this regard, the house fly, *Musca domestica* Linnaeus 1758 (Diptera: Muscidae), is of particular medical importance. As a cosmopolitan, highly synanthropic species intimately associated with decomposing organic matter, feces, and other microbe-rich substrates (Nayduch et al. 2023) it serves as a notorious mechanical vector for a vast reservoir of pathogens, including diverse bacteria, viruses, and parasites (Sasaki et al. 2000; Calibeo-Hayes et al. 2003; Jacques et al. 2017; Geden et al. 2021). Crucially, the capacity of these acquired microbes to establish, proliferate, or persist within the insect’s digestive tract or on its cuticle is highly pathogen-specific. Consequently, certain microbial strains can only be detected transiently or for a limited duration following ingestion, depending on the vector’s internal physiological constraints (Joyner et al. 2013; Fleming et al. 2014).

Such adventitious exsudatophagy may be highly critical during body art procedures. The rapid, repeated needle punctures intensely disrupt the epidermal barrier, creating an open lesion that is highly susceptible to external microbial inoculation, increasing the overall risk of acute cutaneous or soft-tissue infections (Long and Rickman 1994; Bălăceanu-Gurău et al. 2024). Despite the well-known vector competence of synanthropic filth flies, their direct interaction with active tattoo wounds has not yet been documented.

This is the first report of opportunistic exsudatophagous feeding by *M. domestica* on a fresh tattoo wound. We describe the environmental triggers of infiltration, analyze the host–vector interactions during the procedure, and follow the subsequent clinical wound-healing process until complete recovery, supplemented by targeted photographic documentation after 6 and 12 days.

## Materials and methods

This case report is based on a non-interventional observational event conducted in a semi-urban municipality in North Rhine-Westphalia, Germany, on June 10, 2026. Data and specimens were collected during a professional body art tattooing procedure performed on a 32-year-old male client. The observation was made and specimens were collected because one of the authors was present during the procedure as a personal acquaintance of the client, allowing for immediate real-time documentation and sample preservation. Environmental parameters, including ambient outdoor temperature, were obtained from local meteorological records. The structural format of the case report and the applied terminology were adapted from Klüber and Hetz (2026). Photographic documentation of the wound site and subsequent healing progress was performed using a smartphone (Galaxy S25+, Samsung, Suwon, South Korea) on the day of the event as well as 6 and 12 days following the procedure. Written informed consent for the publication of clinical details and photographic material was obtained from the client.

Following capture, the insect specimens were transferred to a 50 mL centrifuge tube and stored at -20 °C until further processing. Subsequent morphological investigations for precise species identification and sex determination, as well as digital imaging, were performed using a VHX-7000 digital microscope (Keyence, Osaka, Japan). The photos were overlaid using GIMP (version 3.2.4, GIMP Development Team) and cropped to the same size. Where necessary, the exposure was adjusted. The figures were rendered using OriginPro 2026 (version 10.3.0.197, OriginLab, Northampton, MA, USA). Morphological identification keys provided by standard dipterological literature were applied to confirm diagnostic features (McAlpine et al. 1981; Marshall et al. 2017).

## Results and discussion

### Client background and procedural settings

The client was a healthy 32-year-old male with no known history of wound healing disorders or systemic metabolic diseases, though presenting with mild localized keratosis pilaris. Due to chronic pain management, the client reported regular intake of ibuprofen, and a daily dose of bilastine was maintained to manage multiple seasonal allergies. The tattooing procedure involved a large-scale artistic tattoo measuring approximately 25 × 10 cm, located on the anterior and medial aspects of the right lower leg, extending from the medial malleolus (inner ankle) to the mid-calf.

The tattoo artist strictly adhered to all legal hygiene protocols and sanitization guidelines, using sterile single-use equipment and maintaining a clean field throughout the procedure. The tattoo studio was located on the ground floor of a multi-tenant building within a semi-urban residential area. However, the studio sat at the ecological periphery of the settlement, approximately 200 m adjacent to agricultural fields. Due to high summer temperatures on the day of the incident, the main entrance door, a back door, and an adjacent window were kept open to facilitate cross-ventilation and fresh air supply, thereby breaching the indoor physical insect barrier.

### Entomological observation and host-vector interactions

**Fig. 1:**
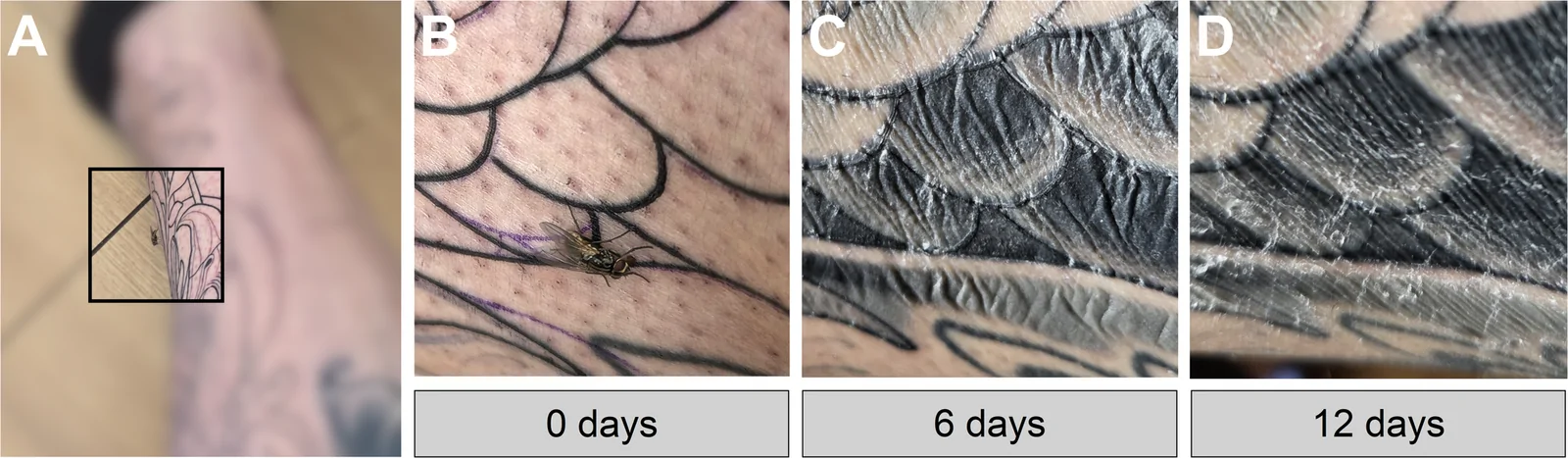
Documentation of an opportunistic feeding event by *M. domestica* during an active tattooing session. **A** Overview of the right lower leg demonstrating an adult house fly specimen settling on the fresh linework of the tattoo wound, with the active feeding site highlighted within a rectangular frame. **B** Close-up view capturing the precise positioning of the fly utilizing its sponging mouthparts to actively drink wound exudates from the fresh tattoo line. Subsequent stages of epidermal healing and desquamation at the identical wound site documented after 6 days (**C**) and 12 days (**D**)

The incident occurred during peak insect activity hours between 12:00 p.m. and 4:00 p.m. (Semakula et al. 1989; Kirse et al. 2025), at a mean ambient outdoor temperature of 26 °C. A total of four adult flies entered the studio through the open entryways and were instantly attracted to the active tattooing site. The flies exhibited highly persistent, repetitive landing attempts directly on the freshly wounded skin barrier. Notably, we observed initial attraction as soon as the epidermal outlines (lining) were breached, but the landing frequency and persistence intensified significantly once extensive shading and solid color gradients were worked into the dermal tissue, resulting in a larger open, exuding surface area (Fig. 1).

The flies attempted to settle on the wet wound tissue during active needle penetration as well as during the 10-to-15-minute procedural breaks scheduled every two hours, during which the leg was left temporarily uncovered (Fig. 1A–B). Constant, active mechanical defensive movements (arm waving) by both the tattoo artist and the immobilized client were required to repel the insects. The flies demonstrated high site-fidelity, immediately returning to the leg wound after being waved off.

### Specimen collection and taxonomic identification

**Fig. 2:**
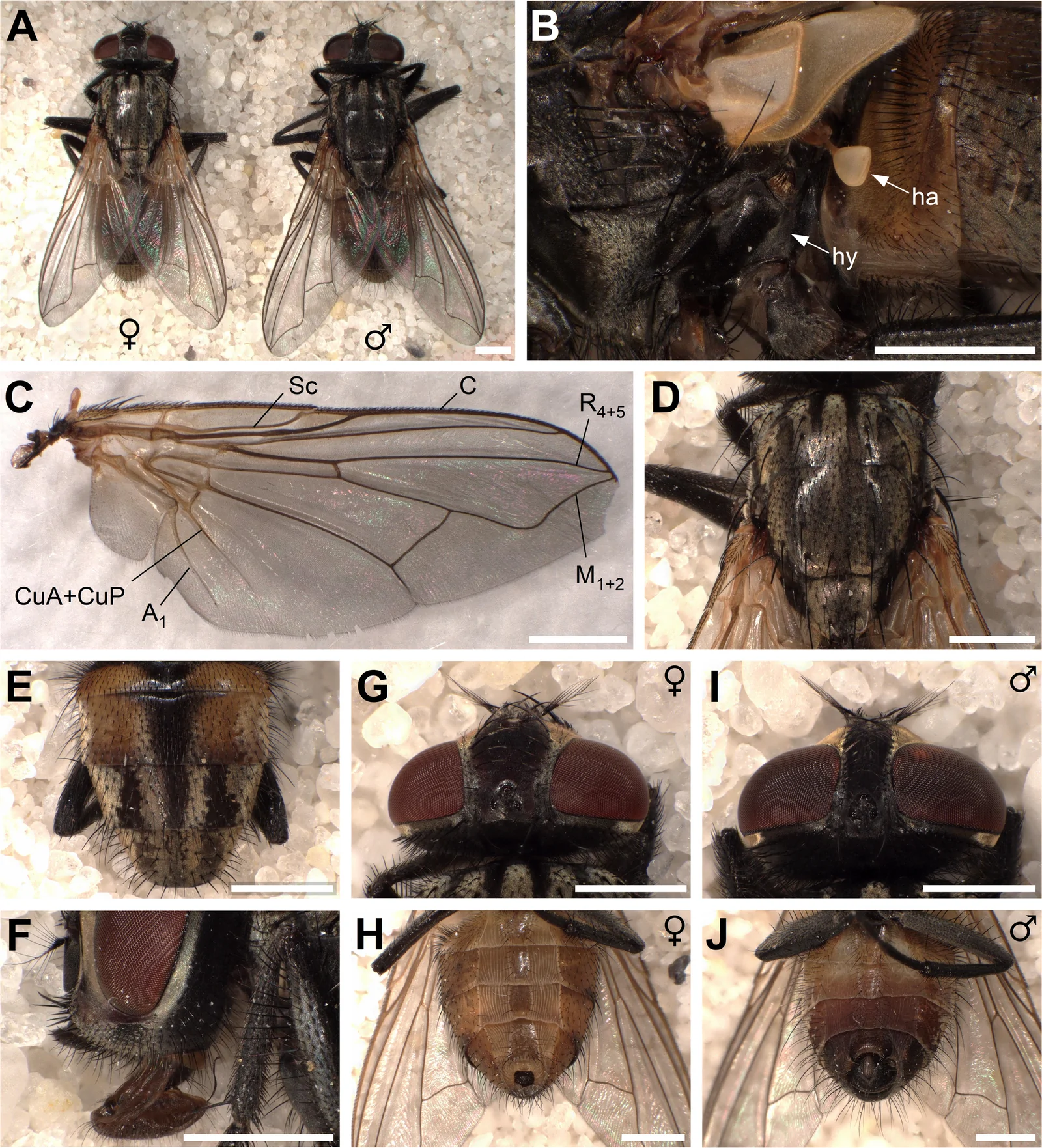
Morphology of *M. domestica* adults. **A** Representative female (left) and male (right) specimens. **B** Lateral aspect of the thoracic region with the haltere (ha) and the hypopleuron (hy) lacking vertical bristles. **C** Ventral view of the left wing with highlighted venation. Dorsal view of (**D**) the mesonotum and (**E**) the abdomen. **F** Laterocranial close-up of the mouthparts. Dorsocranial aspect of (**G**) female and (**I**) male interocular space. Ventrocaudal view of (**H**) female and (**J**) male terminal genitalia (white scale bars = 1 mm)

We successfully captured or neutralized all four interfering insects during the session; individuals that settled on nearby studio surfaces were captured alive using a glass vessel, while flying individuals were eliminated using a mechanical fly swatter. Taxonomic identification under the digital microscope confirmed all specimens as members of the order Diptera, characterized by a single pair of functional membranous forewings (Fig. 2A) and a pair of highly modified, club-shaped hindwings reduced to halteres serving as gyroscopic balancing organs (Fig. 2B; McAlpine et al. 1981).

On a family level, the specimens were assigned to Muscidae. The morphologically similar calyptrate families Calliphoridae and Sarcophagidae were definitively excluded based on the complete absence of a vertical row of distinct bristles on the meron (hypopleuron), a character state that is consistently present in those taxa (Fig. 2B). To ensure complete taxonomic rigor, a systematic differentiation from the closely related muscoid families Anthomyiidae and Fanniidae was performed. The family Anthomyiidae was unequivocally excluded because the first anal wing vein (A_1_) was incomplete and faded out well before the posterior wing margin, whereas a complete vein A_1_ reaching the actual wing margin is a diagnostic feature for Anthomyiidae. The family Fanniidae was subsequently excluded based on the diagnostic configuration of both the A_1_ and subcostal (Sc) veins. In the retrieved specimens, A_1_ vein was not curved forward on a trajectory that would intersect vein CuA + CuP if extended, a critical character state that rules out Fanniidae (where A_1_ is strongly curved forward towards the cubital branch). Furthermore, the Sc vein in the specimens followed a distinctly curved, sinuous trajectory towards the costal vein (C), lacking the straight trajectory characteristic of the Fanniidae (Fig. 2C; Gregor et al. 2002; Marshall et al. 2017).

Following the family assignment, individuals were identified as the house fly, *Musca domestica*, by a combination of diagnostic generic and specific features: the dorsal mesonotum exhibited four characteristic dark, symmetrical longitudinal stripes (Fig. 2D), the abdomen was greyish-yellow and displayed irregular lateral buff-colored markings on the anterior segments alongside a dark median longitudinal stripe (Fig. 2E), and the fourth longitudinal wing vein (M_1 + 2_) showed a prominent, sharp forward bend towards the radial vein (R_4 + 5_) (Fig. 2C). Wing venation and mouthpart anatomy excluded the hematophagous stable fly, *Stomoxys calcitrans* Linnaeus, 1758, which shares a similar size and a quadristriate mesonotum. The retrieved specimens possessed a fleshy, non-piercing, retractile sucking proboscis equipped with prominent labellar lobes and pseudotracheae, anatomically optimized for fluid absorption and exsudatophagy, rather than a rigid, forward-projecting piercing proboscis (Fig. 2F; Lane and Crosskey 1993; Grzywacz et al. 2017; Marshall et al. 2017).

Sex determination based on the evaluation of the interocular space and terminal genitalia (Fig. 2H, J) confirmed the presence of one female individual (Fig. 2G; dichoptic eyes, widely separated) and three male individuals (Fig. 2I; holoptic eyes, closely approximated).

### Clinical progression and wound healing

The insects utilized their specialized sponging mouthparts to actively absorb host fluids directly from the open lesion (Fig. 1B), which consisted of a complex matrix of blood, interstitial fluids, plasma, and excess tattoo ink. Immediately following the completion of the tattooing session, the fresh wound was covered with sterile wound dressings and wrapped in plastic cling wrap for the initial 24 h to re-establish a temporary physical barrier. Afterward, the dressings were carefully removed, and a routine tattoo aftercare protocol was strictly maintained by the client; this included daily gentle washing with lukewarm water and pH-neutral, fragrance-free soap.

Despite the persistent contact with the unsterilized dipteran vectors, the subsequent dermal course was entirely regular. Photographic documentation after 6 and 12 days demonstrated typical superficial epidermal peeling and desquamation without heavy crust formation. The skin initially shed in large flakes (Fig. 1C), followed by the characteristic formation of a shiny, thin “silver skin” before transitioning into a phase of progressively smaller, finer scales, which is highly characteristic of the physiological wound healing process of tattooed skin (Fig. 1D). The large-scale lesion healed completely on an epidermal level after approximately 5 weeks, presenting without any localized skin irritation, persistent erythema, pustule formation, or any clinical signs of secondary bacterial infection or systemic inflammation.

### Epidemiological significance, pathogen risks, and preventative strategies

The entomological observations document an instance of opportunistic exsudatophagy by *M. domestica* during an active body art procedure. While adventitious feeding by non-hematophagous insects is occasionally reported in vulnerable, bedridden patients (Lin et al. 2004; Klüber and Hetz 2026), its documentation on a conscious individual during a professional artistic intervention represents a novel scenario.

The intense attraction of both female and male house flies to the lower leg was driven by volatile organic compounds emitted by the fresh tissue trauma. Tattooing induces broad-surface epidermal disruption, creating an open lesion that continuously exudes blood, interstitial fluid, plasma, and excess ink (Bălăceanu-Gurău et al. 2024). To a cosmopolitan synanthrope like *M. domestica*, with a broad saprophagous feeding spectrum, these protein-rich fluids might serve as an ideal nutritional substrate. The behavioral escalation from initial linework to heavy shading reflects the expanding surface area of exuding tissue, which exponentially increased chemical attractants and drove high site-fidelity.

The saprophagous and coprophilic ecology of *M. domestica* directly underlies its notorious capacity as a mechanical pathogen vector. House flies preferentially feed and breed on contaminated substrates like livestock manure, feces, decaying carcasses, and refuse (Imai 1984; Thomson et al. 2017; Geden et al. 2021; Mupfumi et al. 2026). Constantly alternating between filth and human spaces, their tarsi, cuticular hairs, and mouthparts become heavily loaded with pathogens. In fact, field-collected house flies have been shown to harbor more than 200 distinct microbial species (Nayduch and Burrus 2017), with up to 50% of the species carried by a single individual potentially classifying as pathogenic (Greenberg 1973). Among the bacterial taxa carried, opportunistic pathogens such as *Staphylococcus aureus*, *Pseudomonas aeruginosa*, and several enterobacteria (including *Salmonella* spp., *Shigella* spp., and *Escherichia coli* O157:H7) pose the highest clinical risk (Nayduch and Burrus 2017). Inoculating these vectors into a fresh tattoo site creates a direct pathway for dermal infection, potentially triggering pyogenic skin and soft-tissue infections, including cellulitis or secondary bacterial dermatitis.

This hazard is amplified by the feeding physiology of the house fly. To ingest fluids, *M. domestica* relies on sponging mouthparts, executing cuticular grooming, frequent defecation, and repetitive regurgitation of putative infectious digestive fluids (“bubbling” of regurgitate) directly onto the tissue (Sasaki et al. 2000; McGaughey and Nayduch 2009; Jacques et al. 2017; Geden et al. 2021). Similar exsudatophagous exploitation of sores or open wounds by muscoid dipterans, such as *Musca sorbens* Wiedemann, 1830, has been documented. In addition, *M. domestica* has been described as an eye-seeking fly, opportunistically feeding on tears and eye secretions (Emerson et al. 2000).

Despite intensive exposure, the client experienced an uneventful healing progression, with regular silver-skin formation and desquamation concluding after 5 weeks. This benign outcome is attributable to the artist’s professional sanitization and the client’s rigorous routine aftercare. Immediate application of sterile dressings, followed by daily washing with pH-neutral, fragrance-free soap, likely prevented potential pathogen attachment before a biofilm could establish.

Finally, this case underscores a distinct behavioral vulnerability because the client is subject to temporary physical helplessness during a body art procedure. To ensure precise line application, the client must remain immobilized and actively suppress withdrawal reflexes under pain. This enforced immobility restricts effective mechanical defense, such as swatting, and mimics the physical helplessness of clinical patients.

While direct microbiological isolation from the captured flies or wound exudate was not performed in this observational case report, the established vector competence of *M. domestica* underscores the potential transmission risks. Although federal law and state-level hygiene ordinances in Germany strictly mandate protocols for skin disinfection and instrument sterilization in body art facilities, specific requirements for structural vector barriers are currently lacking. To mitigate these risks during summer, tattoo facilities should install simple mesh fly screens on open entryways to maintain an insect-free workspace. Furthermore, integrating air conditioning units allows studios to keep windows closed, maintaining an uncompromised physical building envelope. Finally, covering the wound with sterile drapes during procedural breaks ensures that exuding tissue is never left exposed.

## Data Availability

All data supporting the findings of this study are available within the paper.
